# Mifepristone Directly Disrupts Mouse Embryonic Development in Terms of Cellular Proliferation and Maturation In Vitro

**DOI:** 10.3390/toxics9110294

**Published:** 2021-11-05

**Authors:** Yu-Ting Su, Jia-Shing Chen, Yi-Ru Tsai, Kuo-Chung Lan, Cheng-Chun Wu, Fu-Jen Huang

**Affiliations:** 1 Department of Obstetrics and Gynecology, Kaohsiung Chang Gung Memorial Hospital, Chang Gung University College of Medicine, Kaohsiung 833, Taiwan; kimyy9487@cgmh.org.tw (Y.-T.S.); lankuochung@gmail.com (K.-C.L.); 2 School of Medicine for International Students, College of Medicine, I-Shou University, Kaohsiung 824, Taiwan; jasonchen1228@isu.edu.tw; 3 An-Ten Obstetrics and Gynecology Clinic, Kaohsiung 802, Taiwan; sexnhappiness@gmail.com; 4 Graduate Institute of Clinical Medical Sciences, Chang Gung University, Kaohsiung 833, Taiwan; 5 Center for Menopause and Reproductive Medicine Research, Kaohsiung Chang Gung Memorial Hospital, Chang Gung University College of Medicine, Kaohsiung 833, Taiwan; 6 School of Medicine, College of Medicine, I-Shou University, Kaohsiung 824, Taiwan; 7 An-An Women and Children Clinic & ART Center, Kaohsiung 807, Taiwan

**Keywords:** mifepristone, endometrium, embryo, development, progesterone, abortion

## Abstract

Mifepristone (RU-486), a synthetic steroid with potent antiprogestogen and anti-glucocorticoid properties, has been widely used in clinical practice. Its effect on the endometrium, ovary, and fallopian tube has been well reported in many human and animal studies. However, its direct impact on post-implantation embryos remains underexplored. Additionally, some women choose to keep their pregnancy after mifepristone treatment fails. Thus, the potential risk remains controversial. Hence, this study investigated the direct effects of mifepristone on the development of mice blastocysts in vitro in terms of implantation and post-implantation. We detected the level of progesterone (P4) associated with ovulation in vivo. The presence of progesterone receptors (PRs) in blastocysts and post-implantation embryos was also evaluated. Cultured embryos were treated directly with mifepristone. We further examined embryonic implantation and post-implantation of blastocysts in vitro to evaluate the direct effects of mifepristone on embryos by the assessment of embryonic outgrowth and differential cell staining. In the oviduct lumen, the P4 level dramatically increased at 48 h and slightly decreased at 72 and 96 h following ovulation. PR was expressed in blastocysts not only in the preimplantation stage but also in the early post-implantation period. In the evaluation of developmental stages, mifepristone significantly reduced the successful ratio of developing into the late egg cylinder and the early somite stage. In addition, it further decreased the cell number of the embryos’ inner cell mass and trophectoderm. We herein provide evidence that mifepristone affects blastocyst viability directly and inhibits post-implantation embryo development in vitro. Furthermore, our data reveal a potential risk of fetus fatality and developmental problems when pregnancies are continued after mifepristone treatment fails.

## 1. Introduction

Mifepristone (RU-486), a synthetic steroid with potent anti-progestogen and anti-glucocorticoid properties, was synthesized by the French company Roussel-Ulcaf in 1980. Since then, it has been widely used in clinical practice [1,2,3] for various applications, mainly including contraception by affecting ovulation [4,5,6,7,8,9] and causing early termination of pregnancy through interfering with embryo implantation [10,11]. However, mifepristone treatment exhibits a limited therapeutic window that lasts <9 weeks of gestation; the incidence of abortion failure increases along the gestational course [12,13]. Furthermore, some women may change their minds and choose to continue the pregnancy after abortion failure [14,15]; however, the potential risks and underlying mechanisms of this are still poorly understood.

The mechanisms underlying the clinical effects of mifepristone remain unclear. The effects of mifepristone on the endometrium have been well reported [16,17,18,19,20,21]. In vivo, it causes changes in the levels of IL-8 and TNF-α and leads to increased progesterone receptor (PR) concentrations in the human fallopian tube [22,23], which may alter the peri-implantation environment and influence fertilization. Animal studies have shown that mifepristone-induced endometrial changes include changes in endometrial secretions and luminal compartments, which adversely affect the growth and viability of pre-implantation embryos [24,25,26,27]. Although previous data have shown that the embryos present with PRs beginning at the blastocyst stage [28], few studies have evaluated the direct effects of mifepristone on post-implantation embryos under conditions independent of the endometrial microenvironment [29].

By culturing blastocysts from superovulated mice, the present study examined the direct effect of mifepristone on embryonic implantation, viability, proliferation, and maturation. This approach has been previously used for analyzing the survival and development of embryos [30,31,32]. This study aimed to assess the temporal and spatial level of P4 in pregnant mice, determine the expression of PR during embryonic development, and to evaluate the direct effect of mifepristone exposure during development in vitro.

## 2. Materials and Methods

### 2.1. Animal and Blastocyst Collection

All animal experiments were approved by the Institution of Animal Care and Use Committee of Kaohsiung Chang Gung Memorial Hospital (No. 2018120702, 10 September 2019) and all animals were cared under humane animal rights according to the Guidelines for Care and Use of Experimental Animals (Council of Agriculture Executive Yuan, Taiwan). ICR virgin albino mice, male mice and pregnant mice were maintained under a 12 h day/12 h night cycle, with food and water available ad libitum. ICR mice (6 ± 8 weeks old) were super-ovulated by injecting 5 IU pregnant mare’s serum gonadotropin, followed by an injection of 5 IU HCG 48 h later. Females were then mated overnight with a single fertile male of the same strain. Pregnancy was confirmed by the presence of a vaginal plug the following day. The next morning after mating, we moved apart the mice with vaginal plugs. We collected the blastocysts by using EBSS medium (0.3% BSA, 1 mM pyruvate sodium, 1 mM glutamine, 2% penicillin/streptomycin) to flush the fallopian tubes on the day 4 morning after the plug was confirmed. We collected the blastocysts using an uncoated 4-well dish and washed it at least three times for the following culture in vitro.

### 2.2. Blastocyst Culture and the Definition of Developmental Stages

The procedures for the acquirement of embryos followed a previous protocol [33,34]. Briefly, blastocysts were acquired by flushing the uterine horn on day 4. Expanded blastocysts from different female mice were pooled and randomly distributed for experiments. The embryos were cultured in Earle’s balanced salt solution (EBSS; Sigma St. Louis, MO, USA) using 0.3% bovine serum albumin (Sigma) during the pre-implantation stage and using CMRL 1066 medium (Sigma) during the post-implantation stage. These mediums contained glutamine (1 mM), sodium pyruvate (1 mM), penicillin (50 IU/mL) (Gibco, Grand Island, NY, USA), and streptomycin (50 mg/mL) (Gibco). CMRL 1066 was applied, including 20% FBS (Gibco) in the culture. We evaluated the embryonic development in a series of stages, including Witschi stages 6–15, following our previous reports [33]; accordingly, we defined embryos which reached stage 9 or 10 by day 4 as early egg cylinder embryos; embryos which reached stage 11, 12 or 13 by day 6 of culture as late egg cylinder embryos; and embryos which reached stage 14 or 15 by day 8 as early somite embryos.

### 2.3. Detection of P4 Level from Serum, Oviduct, Uterus by Radioimmunoassay

To detect the P4 level in serum, venous blood sampling was conducted along the time course following superovulation at 0, 24, 48, 72, and 96 h. To detect the P4 level in the lumen of the oviduct and uterine cavity, respectively, the uterus and oviduct were separated from the sacrificed mice. An equal volume of normal saline was used to flush the lumen of the oviduct and uterine cavity for detecting the P4 level from the collected buffer. Radioimmunoassay was used to detect the P4 level by a commercial assay kit from MyBiosource (San Diego, CA, USA) under the direction of the manual.

### 2.4. Immunofluorescent Staining for PR Receptor

We collected embryos from different developmental stages and fixed the embryos using 4% paraformaldehyde for 30 min at room temperature. We used PBS containing 0.5% Triton X-100 and 1% bovine serum albumin (BSA) to permeabilize the embryos; embryos were then washed three times using PBS containing 0.2% Triton X-100 and 0.3% BSA. We used PBS containing 4% BSA and 0.2% Triton X-100 for blocking the samples at room temperature for 1 h and subjected the sample to hybridization with a primary P4 receptor antibody (Thermo Scientific, Waltham, MA, USA) using a titer of 1:100 overnight at 4 °C. The fluorescein-labeled goat anti-rabbit IgG secondary antibody (31635) was purchased from Thermo Scientific using a titer of 1:100 at room temperature for 1 h. Bisbenzimide (BIS) was counter-stained for nuclear labeling.

### 2.5. Blastocyst Outgrowth Assays

After exposure in RU-486 or vehicle for 24 or 48 h, we collected samples for the embryonic outgrowth assay. The culture medium was carefully removed and replaced by 5% hypotonic sodium citrate (30 μL/well) at room temperature for 5 min [35]. This solution was evaporated under partial vacuum (200 bar) at 50 °C for 60 min. The expanded cells were fixed by FixDenat fixative at 50 °C for 60 min. The total number of nuclei in the outgrowths was examined by a 4% Giemsa staining solution (Sigma) at room temperature for 15 min.

### 2.6. Differential Staining in Blastocysts

After exposure in RU-486 or vehicle for 24 or 48 h, we collected the samples for differential staining [32]. Briefly, we removed the zona pellucida by EBSS medium containing 0.4% pronase and 0.1% BSA. We subjected the denuded blastocysts to exposure to 1 mM of TNBS in a BSA-free M2 medium (M2) containing 0.1% PVP at 4 °C for 30 min. Next, we treated the samples with 30 g/mL of anti-DNP-BSA complex antibody in M2-BSA at 37 °C for 30 min and incubated the samples in M2 supplemented with 10% whole guinea pig serum (GPC, as a source of complement), 20 ug/mL bisbenzimide and 10 ug/mL propidium iodide (PI) at 37 °C for 30 min. The recognition of ICM and TE cells was dependent on the impermeability of the TE layer for propidium iodide (PI) staining. We evaluated the proliferation of blastocysts by calculating the cell numbers from ICM and TE. The proliferation of blastocysts was evaluated by counting the cell number of ICM and TE identified following the procedure of differential staining [32].

### 2.7. Statistical Analysis 

Data were further analyzed using one-way analysis of variance (ANOVA) and *t*-tests. The results were presented as mean ± SEM. The *p*-values less than 0.05 were considered as statistically significant.

## 3. Results

### 3.1. Progesterone Level Correlates with the Time Course of the Preimplantation Stage during Embryonic Development in the Serum, Lumen of the Oviduct, and Uterine Cavity

**Figure 1:**
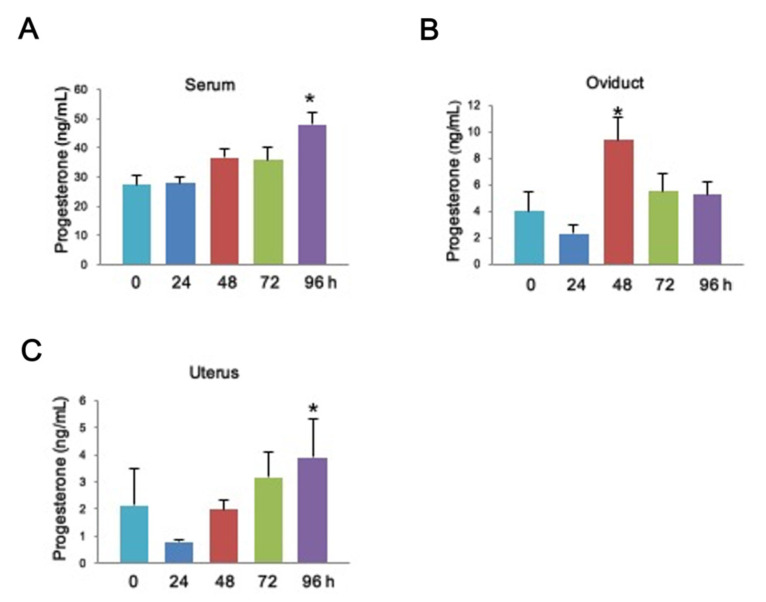
Progesterone levels in the serum, oviduct and uterus correlated with the time course of post-ovulation in female mice. Female mice P4 levels were detected in serum (**A**), the lumen of the oviduct (**B**), and the uterine cavity (**C**) by radioimmunoassay at 0, 24, 48, 72, and 96 h following hCG injection. 0 h *n* = 10, 24 h *n* = 17, 48 h *n* = 20, 72 h *n* = 20, 96 h *n* = 20. Data is represented in mean ± SEM. * denotes *p* < 0.05 by one-way ANOVA.

While progesterone affects uterine function and embryo growth, little is known about the P4 level in the uterine environment from a functional perspective during the period of ovulation and preimplantation. To uncover this issue, we induced superovulation in 8-week-old female mice. We evaluated the level of P4 from the serum, oviduct, and uterus by an immunoradiometric assay at 0, 24, 48, 72, and 96 h after induction of superovulation. To characterize the role of the P4 level in embryogenesis, we detected the P4 level in a time course corresponding to ovulation. In the serum and uterine cavity, the P4 level slowly increased and significantly increased at 96 h after induction of superovulation (Figure 1A,C). In contrast, in the lumen of the oviduct, the P4 level dramatically increased at 48 h after induction of superovulation and slightly decreased at 72 and 96 h (Figure 1B). These data suggest that the P4 level is altered in response to ovulation, indicating the essential role of P4 during the preimplantation stage of the blastocyst.

### 3.2. Mouse Embryo Displays PR Protein Expression during the Progress of Blastocyst Implantation and Early Post-Implantation Stage

**Figure 2:**
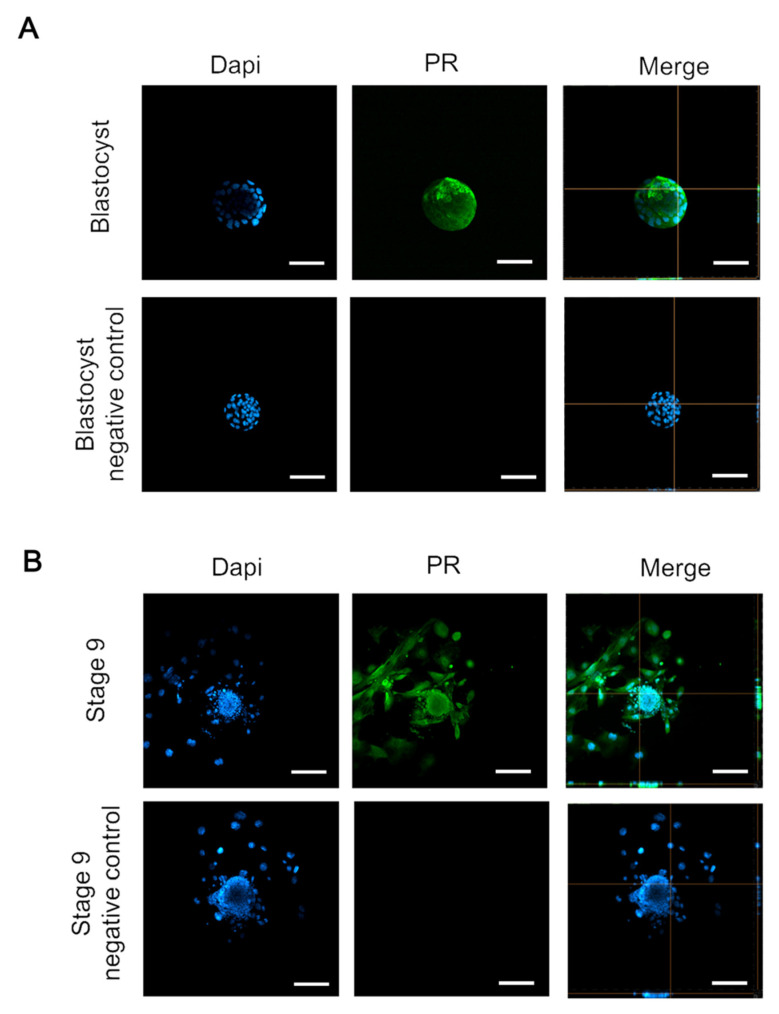
Progesterone receptor protein expression in the embryo from blastocyst to early post-implantation stages. (**A**) Representative immunofluorescent staining for progesterone receptors in the blastocyst stage. DAPI was stained to address the cell nucleus. The negative control was stained using secondary antibody only. Bar: 100 μm. (**B**) Representative immunofluorescent staining for progesterone receptors at the early egg cylinder stage. DAPI was stained to address the cell nucleus. The negative control was stained using a secondary antibody only. Bar: 50 μm.

Although the expression and function of P4 receptor (PR) in the ovary has been reported [36], the expression of PR on embryos during embryogenesis remains controversial. Having confirmed the involvement of P4 during blastocysts’ pre-implantation, we further sought to verify the direct effect of P4 during embryonic development. By immunofluorescent staining of PR, we examined the expression of PR in the peri-implantation and post-implantation phases during embryo development. The data showed that the cultured embryos only presented PR during the developmental stage of blastocysts, but not in the one-, two-, and four-cell and morula stages (Figure S1). By using confocal microscopy, we identified that PR was expressed on blastocysts during the pre-implantation stage and in the early egg cylinder phase during the early post-implantation period (Figure 2A,B), whereas the negative controls exhibited no immunoreactivity. These data suggest that PR may modulate post-implantation embryo development from the initiation of implantation.

### 3.3. Mifepristone Directly Influences the Blastocyst and Early Post-Implantation Stage In Vitro

Having the evidence of PR expressed during embryo development, we next asked whether mifepristone could directly modulate embryonic development during the developmental stages in vitro. We collected and cultured blastocysts from female mice after hCG injection for four days and observed the pattern of implantation and post-implantation of blastocysts, implanted blastocysts, the early and late egg cylinder stage, and the early somite stage in the presence or absence of mifepristone. First, we wondered whether the PR level was affected by mifepristone, but the results were negative (Figure S2). We next examined the direct effect of mifepristone in embryo implantation and maturation using a neutralization approach by treatment with 0.002, 0.2, or 20 μM of mifepristone for 48 h in vitro. The data showed that the implantation rate was not affected by mifepristone in implanted blastocysts. However, in evaluating developmental stages, mifepristone significantly reduced the success rate of development into the late egg cylinder and early somite stages (Table 1). These data suggest that mifepristone directly affects post-implantation embryo development independent of endometrial factors.

### 3.4. Mifepristone Affects Cell Proliferation in the Blastocyst

**Figure 3:**
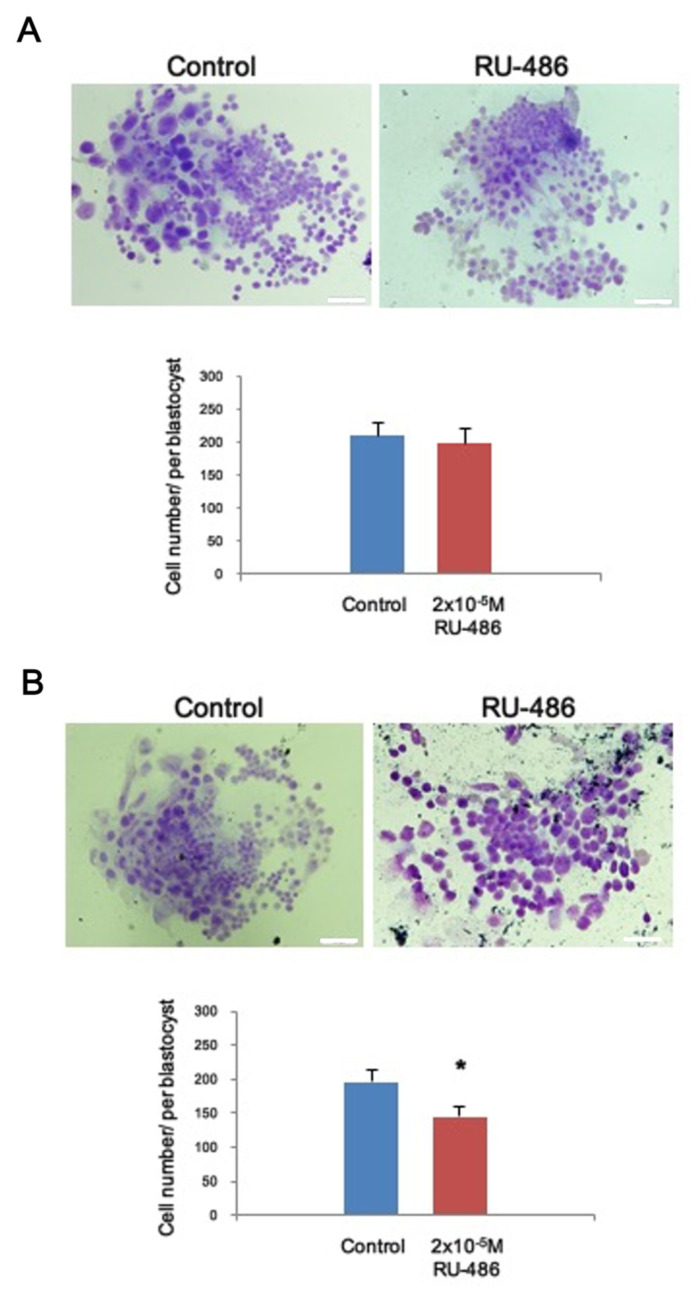
Mifepristone interfered with cell proliferation in the blastocysts. (**A**) Representative image of blastocyst growth following mifepristone exposure for 24 h and (**B**) 48 h. Quantified data from the blastocysts (bottom panel). Control *n* = 50; mifepristone *n* = 45. * denotes *p* < 0.05 by unpaired *t*-test. Bar: 50 μm.

Given that cells can differentiate into inner cell mass and trophectoderm (TE) cells in the blastocyst stage, we thus investigated the effect of P4 on survival/cell proliferation of the blastocysts. In addition, we conducted a time-dependent analysis to examine the direct effect of exposure to 20 μM mifepristone for 24 h (Figure 3A) and 48 h (Figure 3B) on the blastocysts. The data revealed that mifepristone significantly reduced the cell number of blastocysts after exposure for 48 h, resulting in a decrease in the total cell number of blastocysts (Figure 3B).

### 3.5. Mifepristone Affects Blastocyst Outgrowth in the Inner Cell Mass (ICM) and Trophectoderm (TE) In Vitro

**Figure 4:**
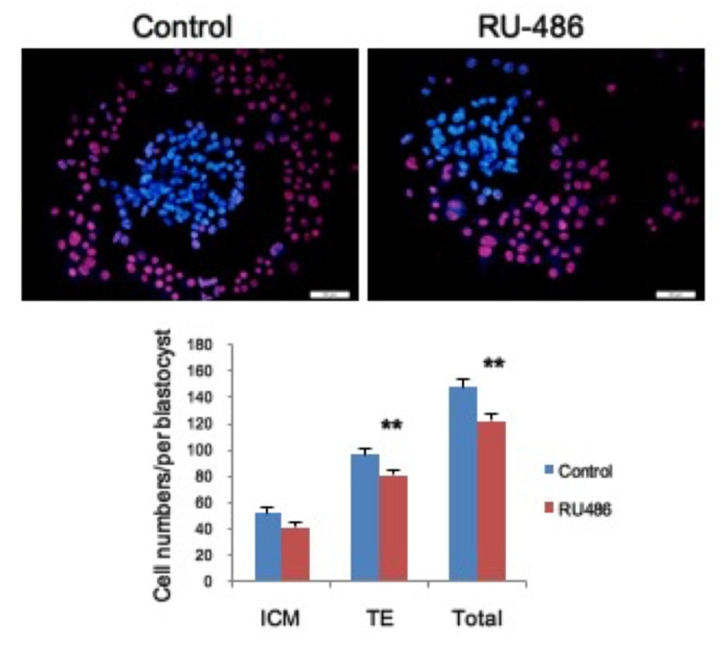
Mifepristone affected cell proliferation in the inner cell mass (ICM) and trophectoderm (TE) during blastocyst outgrowth. Representative differential staining image from cultured blastocysts following exposure in RU-486 for 48 h and followed by culturing for 6 days. Quantified data from the cell number in ICM and TE (bottom panel). Control *n* = 27, mifepristone *n* = 25. ** denotes *p* < 0.01 by unpaired *t*-test. Bar: 100 μm.

Having confirmed the direct modulation of P4 in implanted blastocysts during embryo development, we further examined the effect of P4 in embryonic outgrowth by observing cell proliferation in blastocysts in the ICM and TE. By differential staining, the data indicated that blastocyst outgrowth was slightly decreased in ICM and significantly reduced in TE at eight days in vitro after exposure to mifepristone for 48 h. This data suggests that mifepristone can directly disrupt embryonic outgrowth during the stage of post-implantation (Figure 4).

## 4. Discussion

This study examined the interplay of P4 and P4 receptors in embryogenesis and evaluated the independent effect of mifepristone in embryonic development. Our data confirmed the expression of PR from the pre-implantation stage to the early post-implantation period. In addition, P4 was essential for embryonic development, typically during the stages of implantation and post-implantation; mifepristone exposure resulted in abnormal cell proliferation from the inner cell mass and trophectoderm and impaired blastocyst outgrowth. To our knowledge, we are the first to characterize the direct role of embryonic PR during embryogenesis in vitro. Our data further expand the understanding of P4 in embryo development and medical abortion.

Our data determined that circulating P4 levels vary over the time course and regions in the serum, oviduct, and uterus cavity after ovulation; the increase of P4 was correlated with the time of fertilization and embryo implantation. Furthermore, P4 has been reported to modulate ovulation and preparation of the endometrium for implantation. Thus, the paralleled data from our study are considerable and further support P4 as an essential regulator during preimplantation. In addition, PR can modulate uterus receptivity by inhibiting estrogen-induced epithelial proliferation and activation of P4 target genes, e.g., *Ihh* and *Areg*, to accomplish embryo implantation at pregnancy days 2–3 [37]. Thus, compared to these previous findings, our data further extend the knowledge about the circulating P4 levels in the serum, oviduct, and uterus cavity during preparation for uterine receptivity and preimplantation, supporting the fact that P4 is required in the practice of uterine receptivity.

The contradictory results from several in vitro studies persist in whether P4 is expressed from embryos, as well as whether P4 directly affects embryo development [38,39]. This study herein characterized the expression period of P4 in embryo development. Comparing to previous controversial results from different mammalian species, we found that PR was presented from blastocysts to the early somite stage in mouse embryos and uncovered the direct effects of P4 on the survival and outgrowth of blastocysts. Our findings are correlated to previous reports that the P4 level is critical before embryo implantation [40], which may act directly as a survival factor or indirectly promote the production and secretion of cytokines that contribute to embryonic survival and development [41]. Furthermore, data from in vivo experiments showed that P4 treatment exerts a higher survival and implantation rate in pregnant mice [42], which can activate granulocyte-macrophage colony-stimulating factor secretion from the embryo and endometrium to control embryo survival [43]. Furthermore, this can also increase growth factors produced in the stromal cells [44]. In addition, P4 increases the neural progenitor cell cycle and promotes cell proliferation via progesterone receptor membrane protein 1 and 2 [45]; it is reminiscent of a similar effect during embryo development. Together, comparing these findings from the uterine microenvironment, our data further demonstrated the involvement of P4 in the direct modulation of blastocysts’ survival and outgrowth.

In the present study, our data not only characterized the total cell number of blastocysts affected by mifepristone treatment but also elucidated that TE lineage cells were more vulnerable to mifepristone. TE lineage cells were more affected by mifepristone than ICM cells. Indeed, the primary differentiation event during mammalian development occurs at the blastocysts stage and leads to the delineation of the ICM and TE. Interestingly, a previous study also indicated that TE cells were more sensitive to the octatonic acid-induced impact on embryo growth [46], suggesting that TE is more sensitive to the factors or the microenvironment. In addition, TE cells were more directly exposed in the microenvironment due to their physical distribution in embryos, which may increase the probability of exposure in responded factors. Notably, our data showed RU-486 exposure selectively affected embryo development at the late egg cylinder stage and early somite stage (Table 1). We proposed that the low developmental rate to advanced stages could be explained by the reduced proliferation of the TE cells, which is required to support embryonic development.

Regarding PR downstream signaling, the differentiation and survival of TE and ICM cell lineages are controlled by several factors, including metabolic and signaling pathways, which include WNT, MAPK, NOTCH, integrin-mediated cell adhesion, and PI3K [47]. For instance, PR modulated cell survival and proliferation through the activation of c-Src and downstream MAPK signaling. Furthermore, activation of MAPK also triggers up-regulation of cyclin D1 and entry into the S phase [48]. In addition, PR interacts with PI3K through MAPK signaling [49], and PR can also activate MAPK through membrane PRa and b proteins [50]. These findings are corresponded with our results, suggesting that P4 may independently modulate embryo survival and differentiation via PR or associated proteins without endometrial factors.

In the present study, we found that the TE lineage was more sensitive to PR inhibition than the ICM lineage. Specifically, TE formation was modulated by Ras-MAPK signaling during embryonic development [51], which is also related to PR downstream signaling. While MAPK intracellular signaling was repressed, blastocyst development and TE outgrowth were also altered. Consequently, we speculate that reduced TE cell numbers may result from inhibition of PR-mediated MAPK signaling [52]. On the other hand, regarding ICM lineage formation and survival, Oct 4 is an essential factor in the cell fate decision [53,54]. Furthermore, previous studies have shown that nuclear receptor LRH-1 and COUP TF I/II regulate *Oct4* expression [55,56], and both receptors are involved in PR downstream signaling [57,58]. Consequently, we propose that mifepristone-induced decreases in cell survival and embryo development may result from the PR and its earlier-mentioned downstream signaling pathways; our findings further characterized the direct effect of mifepristone in the survival and differentiation of TE and ICM cell lineage.

Our findings highlight the effects of P4 for basic research of mammalian embryonic development and its implications for medical science and the practice of in vitro fertilization. This study demonstrated that P4 levels are associated with the course of embryogenesis, and embryos can present PR as blastocysts in vitro. Mifepristone can directly disrupt embryogenesis in terms of cell proliferation and developmental maturation. Our data support that keeping a pregnancy after abortion failure by mifepristone may have potential risk.

## Figures and Tables

**Table 1:** In-vitro development of blastocysts following 48-h exposure of mifepristone.

Development (Days In Vitro)	Control Group	Mifepristone Group		
		2 × 10^−9^ M	2 × 10^−7^ M	2 × 10^−5^ M
Blastocysts	101	98	95	102
Hatched/implanted blastocysts (DIV 2)	99 (98.02%)	96 (97.96%)	94 (98.95%)	100 (98.04%)
Early egg cylinder stage (DIV 4)	87 (86.14%)	77 (78.57%)	82 (86.32%)	83 (81.37%)
Late egg cylinder stage (DIV 6)	57 (56.44%)	46 (46.94%)	52 (54.74%)	40 (39.22%) *
Early somite stage (DIV 8)	41 (40.59%)	34 (34.69%)	35 (36.84%)	25 (24.51%) *

* *p* < 0.05 by unpaired *t*-test.

## Data Availability

The data presented in this study are available on request from the corresponding author.

## Supplementary Materials

The following are available online at https://www.mdpi.com/article/10.3390/toxics9110294/s1, Figure S1: The embryos presented progesterone receptor (PR) since the developmental stage of blastocysts. (A) We collected and cultured the mouse embryos in vitro, and performed immunofluorescent staining for PR at stages of 1- cell to blastocysts. (B) The mouse embryos at developmental stages of stage 7–15 presented PR observed by immunofluorescent staining. Bar: 25 μm, Figure S2: RU-486 treatment did not affect the level of progesterone receptor (PR) in mouse blastocysts. We collected the samples for qPCR from cultured blastocysts following exposure in 20 μM of RU-486 for 48 h and followed by culturing for 6 days. Control *n* = 5, RU-486 *n* = 5. Data present in Mean ± SEM.

## References

[1] Glasier A., Thong K., Dewar M., Mackie M., Baird D.T. (1992). Mifepristone (RU 486) Compared with High-Dose Estrogen and Progestogen for Emergency Postcoital Contraception. N. Engl. J. Med..

[2] Parisi C., Steffe M. (1988). A new antiprogestational compound, mifepristone (RU-486). Patol. Clin. Ostet. Ginecol..

[3] Taketani Y., Mizuno M. (1988). Application of anti-progesterone agents for contraception. Rinsho Fujinka Sanka.

[4] Zhu H.-X., Zhang W.-W., Zhuang Y.-L., Huang L.-L. (2009). ORIGINAL ARTICLE: Mifepristone as an Anti-Implantation Contraceptive Drug: Roles in Regulation of Uterine Natural Killer Cells during Implantation Phase. Am. J. Reprod. Immunol..

[5] Wånggren K., Lalitkumar P., Hambiliki F., Ståbi B., Gemzell-Danielsson K., Stavreus-Evers A. (2007). Leukaemia inhibitory factor receptor and gp130 in the human Fallopian tube and endometrium before and after mifepristone treatment and in the human preimplantation embryo. Mol. Hum. Reprod..

[6] Sarkar N.N. (2005). The potential of mifepristone (RU-486) as an emergency contraceptive drug. Acta Obstet. Gynecol. Scand..

[7] Marions L., Danielsson K.G., Swahn M.-L., Bygdeman M. (1998). Contraceptive efficacy of low doses of mifepristone. Fertil. Steril..

[8] Psychoyos A., Nikas G., Sarantis L., Gravanis A. (1995). Hormonal anti-implantation agents: Antiprogestins. Hum. Reprod..

[9] Loutradis D., Bletsa R., Aravantinos L., Kallianidis K., Michalas S., Psychoyos A. (1991). Preovulatory effects of the progesterone antagonist mifepristone (RU486) in mice. Hum. Reprod..

[10] Hsia J.K., Lohr P.A., Taylor J., Creinin M.D. (2019). Medical abortion with mifepristone and vaginal misoprostol between 64 and 70 days’ gestation. Contraception.

[11] Shah D., Rijal P., Thakur A., Rai R. (2018). Mifepristone and Misoprostol Vs Misoprostol Alone in Second Trimester Termination of Pregnancy. J. Nepal Med. Assoc..

[12] Spitz I.M., Bardin C.W., Benton L., Robbins A. (1998). Early Pregnancy Termination with Mifepristone and Misoprostol in the United States. N. Engl. J. Med..

[13] Chen M.J., Creinin M.D. (2015). Mifepristone with Buccal Misoprostol for Medical Abortion: A Systematic Review. Obstet. Gynecol..

[14] Grossman D., White K., Harris L., Reeves M., Blumenthal P.D., Winikoff B., Grimes D.A. (2015). Continuing pregnancy after mifepristone and “reversal” of first-trimester medical abortion: A systematic review. Contraception.

[15] Bernard N., Elefant E., Carlier P., Tebacher M., Barjhoux C.E., Bos-Thompson M.A., Amar E., Descotes J., Vial T. (2013). Continuation of pregnancy after first-trimester exposure to mifepristone: An observational prospective study. BJO Int. J. Obstet. Gynaecol..

[16] Fischer L., Deppert W., Pfeifer D., Stanzel S., Weimer M., Hanjalic-Beck A., Stein A., Straßer M., Zahradnik H., Schaefer W. (2012). Potential hazards to embryo implantation: A human endometrial in vitro model to identify unwanted antigestagenic actions of chemicals. Toxicol. Appl. Pharmacol..

[17] Zhou F., Chen X.-Y., Zhuang Y.-L., Chen Y.-Z., Huang L.-L. (2011). Low-dose mifepristone increases uterine natural killer cell cytotoxicity and perforin expression during the receptive phase. Fertil. Steril..

[18] Sun X., Qiu X., Gemzell-Danielsson K. (2003). Effects of mifepristone on expression of endothelial nitric oxide synthase in human endometrium during the implantation phase. Fertil. Steril..

[19] Qiu X., Sun X., Christow A., Ståbi B., Gemzell-Danielsson K. (2002). The effect of mifepristone on the expression of insulin-like growth factor binding protein-1, prolactin and progesterone receptor mRNA and protein during the implantation phase in human endometrium. Mol. Hum. Reprod..

[20] Danielsson K.G., Swahn M.L., Westlund P., Johannisson E., Seppala M., Bygdeman M. (1997). Effect of low daily doses of mifepristone on ovarian function and endometrial development. Hum. Reprod..

[21] Cameron S.T., Critchley H.O., Buckley C., Kelly R.W., Baird D.T. (1997). Effect of two antiprogestins (mifepristone and onapristone) on endometrial factors of potential importance for implantation. Fertil. Steril..

[22] Li D.-Q., Pan L.-H., Shao Z.-M. (2004). Inhibitory effects of mifepristone on the growth of human gastric cancer cell line MKN-45 in vitro and in vivo. Chin. Med. Sci. J..

[23] Christow A., Sun X., Gemzell-Danielsson K. (2002). Effect of mifepristone and levonorgestrel on expression of steroid receptors in the human Fallopian tube. Mol. Hum. Reprod..

[24] Lalitkumar P.G., Sengupta J., Ghosh D. (2005). Endometrial tumor necrosis factor alpha (TNFalpha) is a likely mediator of early luteal phase mifepristone-mediated negative effector action on the preimplantation embryo. Reproduction.

[25] Sengupta J., Ghosh D. (2000). Role of progesterone on peri-implantation stage endometrium-embryo interaction in the primate. Steroids.

[26] Ghosh D., Nayak N.R., Sengupta J. (1997). Effect of follicular phase administration of mifepristone (RU486) on blastocyst implantation in the rhesus monkey. Contraception.

[27] Ghosh D., Kumar P.G., Sengupta J. (1997). Early luteal phase administration of mifepristone inhibits preimplantation embryo development and viability in the rhesus monkey. Hum. Reprod..

[28] Hou Q., Gorski J. (1993). Estrogen receptor and progesterone receptor genes are expressed differentially in mouse embryos during preimplantation development. Proc. Natl. Acad. Sci. USA.

[29] Lalitkumar P., Lalitkumar S., Meng C., Stavreus-Evers A., Hambiliki F., Bentin-Ley U., Gemzell-Danielsson K. (2007). Mifepristone, but not levonorgestrel, inhibits human blastocyst attachment to an in vitro endometrial three-dimensional cell culture model. Hum. Reprod..

[30] Huang F.-J., Lan K.-C., Kang H.-Y., Liu Y.-C., Hsuuw Y.-D., Chan W.-H., Huang K.-E. (2013). Effect of curcumin on in vitro early post-implantation stages of mouse embryo development. Eur. J. Obstet. Gynecol. Reprod. Biol..

[31] Chang C., Hsuuw Y., Huang F., Shyr C., Chang S., Huang C., Kang H., Huang K. (2006). Androgenic and antiandrogenic effects and expression of androgen receptor in mouse embryonic stem cells. Fertil. Steril..

[32] Huang F., Shen C., Chang S., Wu T.J., Hsuuw Y. (2003). Retinoic acid decreases the viability of mouse blastocysts in vitro. Hum. Reprod..

[33] Hsu Y.-C. (1979). In vitro development of individually cultured whole mouse embryos from blastocyst to early somite stage. Dev. Biol..

[34] Huang F.-J., Wu T.-C.J., Tsai M.-Y. (2001). Effect of retinoic acid on implantation and post-implantation development of mouse embryos in vitro. Hum. Reprod..

[35] Huang F.-J., Chin T.-Y., Chan W.-H. (2013). Resveratrol protects against methylglyoxal-induced apoptosis and disruption of embryonic development in mouse blastocysts. Environ. Toxicol..

[36] Conneely O.M., Lydon J.P., DeMayo F., O’Malley B.W. (2000). Reproductive Functions of the Progesterone Receptor. J. Soc. Gynecol. Investig..

[37] Franco H.L., Rubel C.A., Large M.J., Wetendorf M., Fernandez-Valdivia R., Jeong J., Spencer T.E., Behringer R.R., Lydon J.P., DeMayo F.J. (2012). Epithelial progesterone receptor exhibits pleiotropic roles in uterine development and function. FASEB J..

[38] Salehnia M., Zavareh S. (2013). The Effects of Progesterone on Oocyte Maturation and Embryo Development. Int. J. Fertil. Steril..

[39] Clemente M., de La Fuente J., Fair T., Al Naib A., Gutierrez-Adan A., Roche J.F., Rizos D., Lonergan P. (2009). Progesterone and conceptus elongation in cattle: A direct effect on the embryo or an indirect effect via the endometrium?. Reproduction.

[40] Tian J., Kim S., Heilig E., Ruderman J.V. (2000). Identification of XPR-1, a progesterone receptor required for Xenopus oocyte activation. Proc. Natl. Acad. Sci. USA.

[41] Martal J., ChÊne N., Camous S., Huynh L., Lantier F., Hermier P., L’haridon R., Charpigny G., Charlier M., Chaouat G. (1997). Recent developments and potentialities for reducing embryo mortality in ruminants: The role of IFN-tau and other cytokines in early pregnancy. Reprod. Fertil. Dev..

[42] Ghaemi S.R., Salehnia M., Valojerdi M.R. (2008). The Effect of Progesterone and Exogenous Gonadotropin on Preimplantation Mouse Embryo Development and Implantation. Exp. Anim..

[43] Robertson S.A. (2007). GM-CSF regulation of embryo development and pregnancy. Cytokine Growth Factor Rev..

[44] Lessey B.A., Ilesanmi A.O., Castelbaum A.J., Yuan L., Somkuti S.G., Satyaswaroop P.G., Chwalisz K. (1996). Characterization of the functional progesterone receptor in an endometrial adenocarcinoma cell line (Ishikawa): Progesterone-induced expression of the α1 integrin. J. Steroid Biochem. Mol. Biol..

[45] Liu L., Wang J., Zhao L., Nilsen J., McClure K., Wong K., Brinton R.D. (2009). Progesterone Increases Rat Neural Progenitor Cell Cycle Gene Expression and Proliferation Via Extracellularly Regulated Kinase and Progesterone Receptor Membrane Components 1 and 2. Endocrinology.

[46] Fredrickson J., Krisher R., Morbeck D.E. (2015). The impact of the protein stabilizer octanoic acid on embryonic development and fetal growth in a murine model. J. Assist. Reprod. Genet..

[47] Adjaye J., Huntriss J., Herwig R., Benkahla A., Brink T.C., Wierling C., Hultschig C., Groth D., Yaspo M.-L., Picton H.M. (2005). Primary Differentiation in the Human Blastocyst: Comparative Molecular Portraits of Inner Cell Mass and Trophectoderm Cells. Stem Cells.

[48] Faivre E., Skildum A., Pierson-Mullany L., Lange C.A. (2005). Integration of progesterone receptor mediated rapid signaling and nuclear actions in breast cancer cell models: Role of mitogen-activated protein kinases and cell cycle regulators. Steroids.

[49] Bagowski C.P., Myers J.W., Ferrell J.E. (2001). The Classical Progesterone Receptor Associates with p42 MAPK and Is Involved in Phosphatidylinositol 3-Kinase Signaling inXenopus Oocytes. J. Biol. Chem..

[50] Hanna R., Pang Y., Thomas P., Zhu Y. (2006). Cell-surface expression, progestin binding, and rapid nongenomic signaling of zebrafish membrane progestin receptors alpha and beta in transfected cells. J. Endocrinol..

[51] Lu C.-W., Yabuuchi A., Chen L., Viswanathan S., Kim K., Daley G.Q. (2008). Ras-MAPK signaling promotes trophectoderm formation from embryonic stem cells and mouse embryos. Nat. Genet..

[52] Boonyaratanakornkit V., Scott M.P., Ribon V., Sherman L., Anderson S.M., Maller J.L., Miller W., Edwards D.P. (2001). Progesterone Receptor Contains a Proline-Rich Motif that Directly Interacts with SH3 Domains and Activates c-Src Family Tyrosine Kinases. Mol. Cell.

[53] Takaoka K., Hamada H. (2012). Cell fate decisions and axis determination in the early mouse embryo. Development.

[54] Rossant J., Tam P.P.L. (2009). Blastocyst lineage formation, early embryonic asymmetries and axis patterning in the mouse. Development.

[55] Ben-Shushan E., Sharir H., Pikarsky E., Bergman Y. (1995). A dynamic balance between ARP-1/COUP-TFII, EAR-3/COUP-TFI, and retinoic acid receptor:retinoid X receptor heterodimers regulates Oct-3/4 expression in embryonal carcinoma cells. Mol. Cell. Biol..

[56] Gu P., Goodwin B., Chung A.C.-K., Xu X., Wheeler D.A., Price R.R., Galardi C., Peng L., Latour A.M., Koller B.H. (2005). Orphan Nuclear Receptor LRH-1 Is Required to Maintain Oct4 Expression at the Epiblast Stage of Embryonic Development. Mol. Cell. Biol..

[57] Kurihara I., Lee D.K., Petit F.G., Jeong J., Lee K., Lydon J.P., DeMayo F.J., Tsai M.J., Tsai S.Y. (2007). COUP-TFII mediates progesterone regulation of uterine implantation by controlling ER activity. PLoS Genet..

[58] Labelle-Dumais C., Pare J.F., Belanger L., Farookhi R., Dufort D. (2007). Impaired progesterone production in Nr5a2+/- mice leads to a reduction in female reproductive function. Biol. Reprod..

