# Supplementary material for: Mifepristone Directly Disrupts Mouse Embryonic Development in Terms of Cellular Proliferation and Maturation In Vitro
Source: Toxics. 2021 Nov 5;9(11):294. doi: 10.3390/toxics9110294 (PMC8623230; doi:10.3390/toxics9110294)
Supplement: Supplementary file 1 [file toxics-09-00294-s001.zip › toxics-1434995-supplementary.pdf]

toxics

Supplementary Materials: Mifepristone Directly Disrupts Mouse Embryonic Development in Terms of Cellular Proliferation and Maturation In Vitro

Yu-Ting Su, Jia-Shing Chen, Yi-Ru Tsai, Kuo-Chung Lan, Cheng-Chun Wu and Fu-Jen Huang

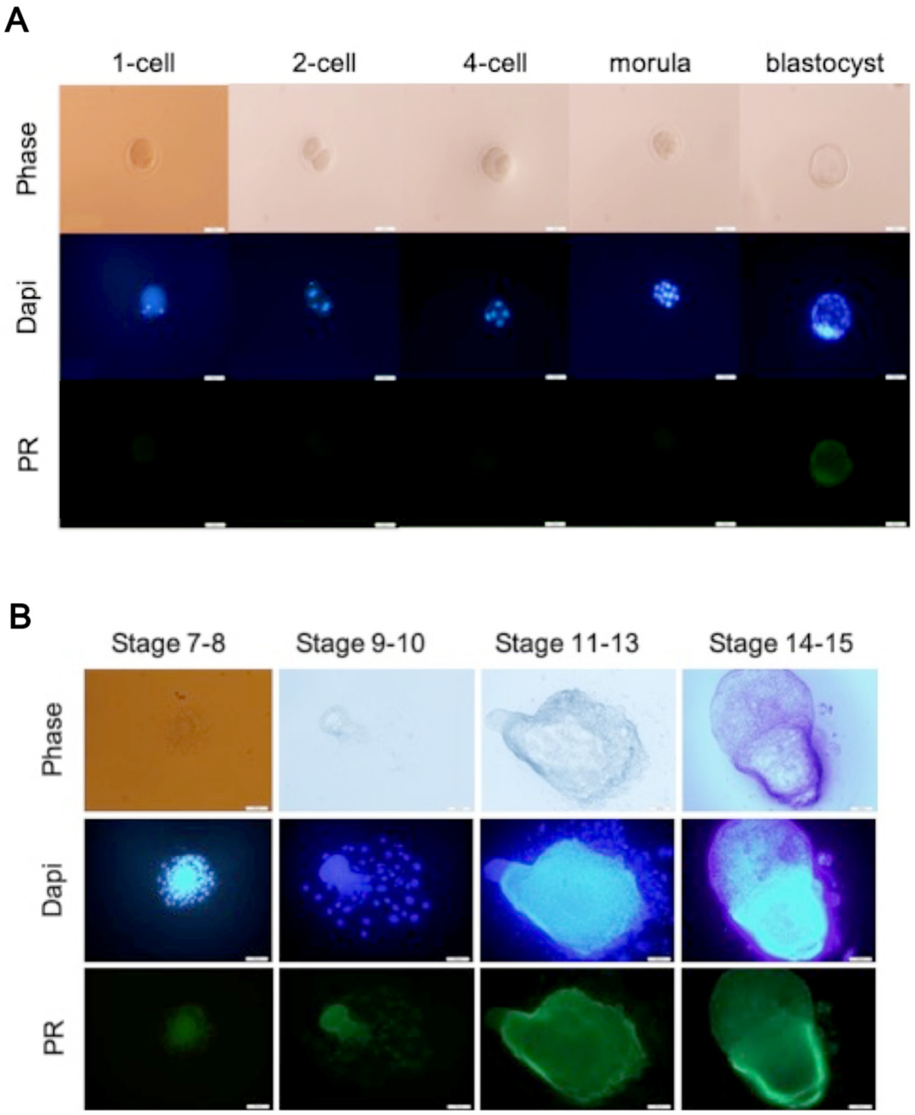

Figure S1. The embryos presented progesterone receptor (PR) since the developmental stage of blastocysts. **(A)** We collected and cultured the mouse embryos in vitro, and performed immunofluorescent staining for PR at stages of 1- cell to blastocysts. **(B)** The mouse embryos at developmental stages of stage 7 – 15 presented PR observed by immunofluorescent staining. Bar: 25 µm.

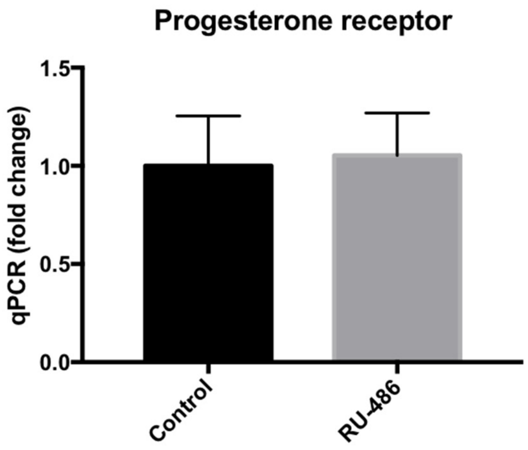

Figure S2. RU-486 treatment did not affect the level of progesterone receptor (PR) in mouse blastocysts. We collected the samples for qPCR from cultured blastocysts following exposure in 20 μM of RU-486 for 48 h and followed by culturing for 6 days. Control *n* = 5, RU-486 *n* = 5. Data present in Mean ± SEM.
